# Reimagining the future of natural history museums with compassionate collection

**DOI:** 10.1371/journal.pbio.3002101

**Published:** 2023-05-04

**Authors:** Allison Q. Byrne

**Affiliations:** 1 Department of Environmental Science, Policy & Management, University of California, Berkeley, California, United States of America; 2 Museum of Vertebrate Zoology, University of California, Berkeley, California, Unites States of America

## Abstract

This Perspective advocates for the adoption of compassionate collection practices, through which natural history museums can better maintain existing collections, accommodate more non-lethal specimens and data, and foster an inclusive community that celebrates the innate connections linking human and non-human lives.

## Teaser

Compassionate collection involves minimizing harm while collecting museum data in the field. By adopting this practice, natural history museums could better maintain existing collections, accommodate more nonlethal specimens and data, and foster an inclusive community.

Natural history museums are essential hubs for research and education. They are also spaces where people can express their wonderment at the natural world in community. Recently, I witnessed my students gazing in awe at the century-old amphibian specimens before them. Preparing to collect swab samples, one student said to another “this museum is my favorite place on campus.” Experiencing biodiversity in a museum is a powerful experience for students. As a museum researcher and educator, I wholeheartedly support ensuring the continuity of these resources going forward. However, I believe it is time to reimagine the natural history museums of the future.

The growth of museum collections through live specimen collection [1] is a relic of the extractive, colonial science practices of the 20th century [2]. While others have already discussed whether collecting might jeopardize threatened wildlife populations [3,4], there is another harm perpetuated through this practice. Removing an animal from its natural habitat and killing it for the purpose of storing it in a museum collection reinforces the stance that humans have dominion over other living creatures. As educators, we have a responsibility to address the way we relate to animals in biodiversity science, fostering the natural connection that attracts many scientists to this field. I propose we adopt a new framework for collection practices that I term “compassionate collection” (Box 1).

**Box 1. What is compassionate collection?**

The practice of compassionate collection involves a reorientation in the way the natural history museum community relates to the animals they collect. By taking a holistic view of the world around us, we can see that we do not need to sacrifice rigorous research to preserve animal lives (and vice versa). Rather, when we center compassion in museum science, we open the door to a more resilient, diverse, and forward-thinking future for natural history museums.

Compassionate collection means:

- Growing museum collections through improved storage and databasing of nonlethal samples such as tissues, recordings, photos, or other individual-based data.
- Maintaining museum collections by investing in infrastructure that prioritizes the longevity of existing specimens and samples.
- Embracing new technologies to unlock the potential of existing collections and optimize new collection practices for emerging research applications.
- Welcoming a new generation of diverse museum scientists by building community around connection and respect for the wildlife around us and the planet that we share.

The concept of compassionate collection borrows from the compassionate conservation movement, which emphasizes avoiding unnecessary harm to individual animals when considering conservation actions [5,6]. Similarly, compassionate collection necessitates minimizing harm to individual creatures while collecting museum data in the field by using techniques such as collecting nonlethal DNA samples, photos, or recordings. Furthermore, this practice emphasizes respecting the lives that have already been sacrificed by prioritizing the maintenance, care, and accessibility of existing collections. This approach also protects the integrity of the collection experience and encourages students to foster connections with their fellow living beings rather than setting these feelings aside in the name of science.

Many would agree that there is immense untapped potential in existing collections. Providing stable financial support and encouraging research on existing collections embodies the ethos of compassionate collection because it honors the lives that have already been taken. Through this lens, preventing the deterioration of existing collections and building and maintaining database infrastructure to make collections accessible is paramount. Recognizing that space, time, and funding are limited in most museum contexts, resources otherwise earmarked for new whole animal collections could be better used in this capacity.

An important part of compassionate collection is the embracing of new technologies, thereby expanding the breadth of collection-based science. From sequencing ancient DNA [7] to large-scale digitization efforts [8], technological advances continue to reveal the hidden value of existing collections. In my own work, I sequence pathogen DNA to trace wildlife disease outbreaks through time. While this retrospective research would not be possible without existing collections, that is not the case for research using new specimen samples. Rather, a well-preserved and catalogued set of skin swabs would serve the same purpose by providing ample host and pathogen DNA. Furthermore, properly preserved nonlethal tissue samples could provide high-quality DNA for myriad future uses. As compassionate collection necessitates preserving the life of an animal and minimizing harm, it will yield less biological material than collecting a whole body. However, as techniques like whole-genome sequencing become the norm, and centralized data sharing continues to grow [9], the need for repetitive sequencing experiments using a single animal will continue to diminish over time.

Technological advancements have opened the door to imagining new futures for natural history museums—ones that value growth to accommodate the “extended specimen,” or the set of physical and digital data associated with an individual [10]. I propose that we continue to use these technologies to replace the need for whole animal bodies. Through this reorientation, we can improve our ability to do science by collecting, storing, and cataloging samples in ways that are already optimized for research applications.

Finally, compassionate collection recognizes the importance of the emotional connection that links human and nonhuman lives. To highlight this experience, let us consider the example of a student joining a collecting field trip. This new scientist is full of enthusiasm, carefully following the directions of the senior team members. The excitement of this trip reaches a climax as the student first encounters the elusive creature they were seeking, marveling at its beauty and behavioral intricacies. Then, the reality sinks in: because they found this creature, it will not live to see another day. Finding the next animal feels different, and they pause before alerting the group. They stop to consider the moral weight of taking a life versus achieving the team’s goals. They wonder if they might not be cut out for this field, a sentiment perhaps directly or indirectly communicated to them by other team members. They begin to believe that compassion is a weakness and disqualifying trait for a museum scientist.

While this scenario is hypothetical, it is informed by the experiences I have had within the museum community. Field work can be a vulnerable time, as it may be in a remote or isolated environment with relative strangers and an established hierarchy. Studies have shown that harmful behavior during field experiences can significantly impact the mental health and career trajectory of trainees [11]. A compassionate collection approach would establish the moral value of animal lives, creating clear expectations for how animals are treated in the field. Critically, it would nurture the love and wonder that is shared by many enthusiastic new scientists and would indicate to them that they do belong in this field.

Reflexivity is a healthy practice. We must continue to ask ourselves, are we adequately serving society and upholding our values? I believe that we can do better, and I offer an empathic framework to consider for collection practices going forward (Box 1). Museums can be repositories of new specimen information, not bodies. Let us expand our ability to accession and loan nonlethal samples. Let us continue to build database infrastructure for sharing photos, scans, recordings, and other individual data. Let us emphasize conducting research on existing collections and applying new technologies to these pursuits. Let us continue to collect salvage animals (i.e., roadkill and mortalities from wildlife rescues): a soberingly plentiful source of new specimens in an era of increasing human–wildlife interactions. Under this framework, museum collections will be a more vibrant resource that is built on empathy, fostering a foundation for a more diverse and welcoming community of compassionate museum scientists.
